# Cesarean Section Trend in a University Hospital in Thailand

**DOI:** 10.1155/jp/8192268

**Published:** 2026-02-25

**Authors:** Dittakarn Boriboonhirunsarn

**Affiliations:** ^1^ Department of Obstetrics and Gynecology, Faculty of Medicine Siriraj Hospital, Mahidol University, Bangkok, Thailand, mahidol.ac.th

## Abstract

**Background:**

An increasing trend of cesarean section (CS) rates has been observed worldwide, including Thailand. Various interventions to decrease unnecessary CS have been implemented as per recommendations. This study is aimed at evaluating a trend in CS rate during a 42‐month period in a university‐based tertiary care hospital in Thailand.

**Methods:**

In a cross‐sectional study, women who delivered at Siriraj Hospital between January 2021 and June 2024 were included. Those with private services were excluded. All women were categorized into 10 groups according to Robson classification. Overall and group‐specific CS rates and their contribution to CS rate were evaluated. Trend of CS rate was evaluated by locally weighted scatterplot smoothing (LOESS) method.

**Results:**

Of 13,645 deliveries, 2868 had private services, leaving 10,777 deliveries included in the analysis. Overall CS rate was 42.9% (95% CI: 42.1%–43.8%) and did not significantly change during the study period. Women in Groups 1 and 5 had the highest CS contribution of 25%–27%. Overall CS rate in Group 1 was 33.9% and the rates did not change significantly but showed a slight decrease to 31.4% in 2024. The LOESS regression showed that overall CS rate slightly increased during 2021, slightly decreased during 2022, and remained relatively stable during 2023 and 2024 at approximately 43%. CS rate in Group 1 slightly decreased during 2021, slightly increased during 2022, and a more obvious decrease during 2023–2024 was observed.

**Conclusion:**

Over a 42‐month period, a relatively stable high overall CS rate of 42.9% was observed, with the highest contribution from women in Groups 1 and 5 of the Robson classification. For women in Group 1, the CS rate showed a notable decrease during 2023–2024.

## 1. Introduction

Although the World Health Organization (WHO) recommends that the cesarean section (CS) rate should be 10%–15% at the population level, an increasing trend of CS rates has been reported in many countries worldwide, including Thailand. [1–6] It is projected that, by 2030, 28.5% of women worldwide will give birth via CS. [3] CSs, when medically indicated, are effective in saving both maternal and infant lives. However, CS can increase many short‐ and long‐term risks to the women and infants, and increased use of unnecessary CS also represents excessive resource utilization without any benefit. [7–9] Therefore, several organizations have advised a variety of clinical and nonclinical strategies to decrease unnecessary CS. [10–14]

The WHO recommends the use of the Robson classification (Table 1) as a standard for assessing, monitoring, and comparing CS rates within and between healthcare facilities over time. [1, 2, 15, 16] The use of the Robson classification will also help in identifying specific groups of women with a high contribution to CS and designing appropriate interventions to decrease the CS rate as well as evaluating the changes.

**Table 1:** Robson classification.

Group	Characteristics
Group 1	Nulliparous with single cephalic pregnancy, ≥ 37 weeks gestation in spontaneous labor
Group 2	Nulliparous with single cephalic pregnancy, ≥ 37 weeks gestation who either had labor induced (2a) or were delivered by cesarean section before labor (2b)
Group 3	Multiparous without a previous uterine scar, with single cephalic pregnancy, ≥ 37 weeks gestation in spontaneous labor
Group 4	Multiparous without a previous uterine scar, with single cephalic pregnancy, ≥ 37 weeks gestation who either had labor induced (4a) or were delivered by cesarean section before labor (4b)
Group 5	All multiparous with at least one previous uterine scar, with single cephalic pregnancy, ≥ 37 weeks gestation
Group 6	All nulliparous women with a single breech pregnancy
Group 7	All multiparous women with single breech pregnancy, including women with previous uterine scars
Group 8	All women with multiple pregnancies, including women with previous uterine scars
Group 9	All women with a single pregnancy with a transverse or oblique lie, including women with previous uterine scars
Group 10	All women with a single cephalic pregnancy < 37 weeks gestation, including women with previous scars

Two WHO multicountry surveys reported that the CS rate in Thailand increased from 34.1% in 2007–2008 to 39.7% in 2014–2015. [2, 17] Siriraj Hospital, which is the largest university‐based tertiary hospital in Thailand, has been using the Robson classification to evaluate the CS rate since 2017. The overall CS rate has been reported to be as high as 48.9% in 2018 and 40.6% by 2023. [18–19] Another university hospital in southern Thailand also reported an overall CS rate of 55.5%. [20] A more recent study in two tertiary care hospitals revealed a CS rate of 45%. [21] As consistently reported, pregnant women in Groups 5 and 1 have the highest contribution to the overall CS rate. However, as of current practice in Thailand, repeat CS will always be performed in women with previous CS unless vaginal delivery is imminent. Therefore, women in Group 1 (nulliparous with single cephalic pregnancy, ≥ 37 weeks gestation in spontaneous labor) were identified as the primary target for the efforts to reduce the CS rate.

Over the past many years, several measures have been gradually implemented as a quality improvement process in order to reduce the CS rate. The interventions were designed in accordance with recommendations, which included an improved educational program for pregnant women (2019), updates to intrapartum guidelines and algorithms (2019), a mandatory second opinion (2019), and an audit and feedback system (2020). Therefore, the objective of this study was to evaluate a trend in CS rate from 2021 to 2024 to observe if there were any changes during the 3.5‐year period. The results would help in better understanding the situation of CS in our institution and in Thailand as well as planning future initiatives to reduce unnecessary CS.

## 2. Methods

After approval from the Siriraj Institutional Review Board (SIRB), a cross‐sectional study was conducted at the Department of Obstetrics and Gynecology, Faculty of Medicine Siriraj Hospital in Bangkok, Thailand. All women who delivered at the hospital between January 2021 and June 2024 were initially assessed. In order to avoid confounding effects on the results, the study excluded pregnant women who received private services since an abnormally high CS rate among this group has previously been reported [22–24] and observed in our institution. Private services are defined as private care provided by a specific obstetric staff of women′s choice, and the women are obliged to provide customary gratitude payments to their private obstetrician. All the women included received standard care according to institutional guidelines by attending in‐training residents in obstetrics and gynecology, under staff supervision. The decision to perform CS was at the attending staff′s discretion.

### 2.1. Changes During the Study Period

Since 2019, many interventions have been gradually incorporated into clinical practice as part of the efforts to reduce unnecessary CS. The interventions have been modified from what was recommended by many organizations. [10, 11, 13]

Appropriate educational intervention for the women has been recommended by the WHO. [10] Previously, two educational classes were provided to all pregnant women, covering the topics of general prenatal care and birth preparation. For the past few years, information on modes of delivery and their benefits and risks has been added into the educational content. The aim was to help women better understand the advantages and disadvantages of CS and make better and more appropriate decisions on their mode of delivery.

Evidence‐based intrapartum care, as recommended by the WHO and the American College of Obstetricians and Gynecologists (ACOG), has been adopted and modified to fit with local context. [11, 13, 14] This included the change of the definition of the active phase of the first stage of labor to 5 cm of cervical dilatation, before which interventions such as amniotomy should be avoided. In addition, the diagnosis of labor arrest should only be made after such a threshold. [11, 13, 14] These changes have been distributed to all in‐training residents and obstetric staff with regular audits to ensure compliance.

During the study period, an audit and feedback system has also been initiated as recommended by the WHO [10] The audit team is primarily composed of obstetricians, labor room nurses, and residents′ representatives. The audit was performed by reviewing randomly selected medical records of women in Groups 1–4 who delivered by CS. After discussion, each case was classified into fulfilled, borderline, and unfulfilled indications for CS. The results were regularly reported during obstetric staff meetings, and constructive feedback was prepared and disseminated.

### 2.2. Data Collection and Statistical Analysis

All included women were categorized according to Robson classification. Overall and group‐specific CS rates and their contribution to the CS rate were calculated. The trend of the CS rate over the study period was evaluated by a scatter plot and locally weighted scatterplot smoothing (LOESS) method. The LOESS kernel Epanechnikov method was used, and the percentage of points to fit was set to 50% to provide smooth curve estimates with the balance between bias and variances, and not to over‐ or underfit the trend. This will help evaluate the trend in the nonlinear relationship between CS rate and time. In addition, it will better create a visual aid for better understanding and interpretation from the smooth curve. All the analyses were performed using IBM SPSS software Version 21.0 (IBM Inc., Armonk, New York).

## 3. Results

During the study period, there were 13,645 deliveries, of which 2868 had private services (21%), leaving 10,777 deliveries included in the analysis. There were 3572, 3337, 3868, and 1531 deliveries in 2021, 2022, 2023, and 2024 (January to June), respectively. CS was performed in 6170 cases, corresponding to an overall CS rate of 42.9% (95% CI: 42.1%–43.8%).

All deliveries in each year were classified according to the Robson classification and the results are shown in Table 2. Overall distributions of the women in each group were similar during the 42‐month period, with the majority of the women in Groups 1 and 3. A significant increasing trend in the proportion of women in Group 10 (preterm delivery) was observed from 10.1% in 2021 to 11.8%, 13.0%, and 12.9% in 2022, 2023, and 2024, respectively (*p* = 0.001).

**Table 2:** Robson classification of all deliveries in 2021–2024.

Group	2021			2022			2023			2024 (January–June)		
	No. of women (%)	CS in group (%)	Relative CS (%)	No. of women (%)	CS in group (%)	Relative CS (%)	No. of women (%)	CS in group (%)	Relative CS (%)	No. of women (%)	CS in group (%)	Relative CS (%)
1	1262 (33.6)	419 (33.2)	26.6	1087 (32.6)	392 (36.1)	27.2	1359 (35.1)	445 (32.7)	27.5	519 (33.9)	163 (31.4)	25.3
2a	88 (2.3)	63 (71.6)	4.0	51 (1.5)	37 (72.5)	2.6	66 (1.7)	38 (57.6)	2.3	46 (3)	31 (67.4)	4.8
2b	49 (1.3)	49 (100)	3.1	54 (1.6)	51 (100)	3.7	45 (1.2)	45 (100)	2.8	17 (1.1)	17 (100)	2.6
3	1199 (32.0)	152 (12.7)	9.7	1091 (32.7)	129 (11.8)	8.9	1117 (28.9)	133 (11.9)	8.2	440 (28.6)	54 (12.3)	8.4
4a	45 (1.2)	15 (62.0)	1.0	21 (0.6)	11 (62.0)	0.8	34 (0.9)	15 (62.0)	0.9	17 (1.1)	6 (62)	0.9
4b	18 (0.5)	18 (100)	1.1	20 (0.6)	20 (100)	1.4	18 (0.5)	18 (100)	1.1	14 (0.9)	14 (100)	2.2
5	461 (12.3)	458 (99.3)	29.1	395 (11.8)	390 (98.7)	27.0	432 (11.2)	428 (99.1)	26.4	167 (10.9)	167 (100)	25.9
6	70 (1.9)	67 (95.7)	4.3	70 (2.1)	67 (95.7)	4.6	82 (2.1)	79 (96.3)	4.9	29 (1.9)	28 (96.5)	4.3
7	76 (2.0)	70 (92.1)	4.4	63 (1.9)	60 (95.2)	4.2	85 (2.2)	82 (96.5)	5.1	33 (2.2)	28 (84.8)	4.3
8	90 (2.4)	70 (77.8)	4.4	71 (2.1)	59 (83.1)	4.1	111 (2.9)	89 (80.2)	5.5	50 (3.3)	43 (86)	6.7
9	16 (0.4)	16 (100)	1.0	20 (0.6)	20 (100)	1.4	15 (0.4)	15 (100)	0.9	1 (0.1)	15 (100)	0.2
10	378 (10.1)	178 (47.1)	11.3	394 (11.8)	204 (51.8)	14.1	504 (13.0)	234 (46.4)	14.4	198 (12.9)	93 (47)	14.4
	3572	1575 (42.0)		3337	1443 (43.2)		3868	1621 (41.9)		1531	645 (42.1)	

Overall CS rate was not significantly different during the study period. The rates were 42%, 43.2%, 41.9%, and 42.1% in 2021, 2022, 2023, and 2024, respectively. For each year, women in Groups 1 and 5 had the highest contribution of all CS (approximately 25%–27% each). Overall CS rate in Group 1 was 33.9%, and the rates did not change significantly, that is, 33.2%, 36.1%, and 32.7% in 2021, 2022, and 2023, but showed a slight decrease to 31.4% in 2024. The CS rate in Group 3 was comparable between different years at approximately 12%. In Group 10, a higher CS rate was observed at approximately 45%–50%, which was not significantly different between each year.

**Figure 1:**
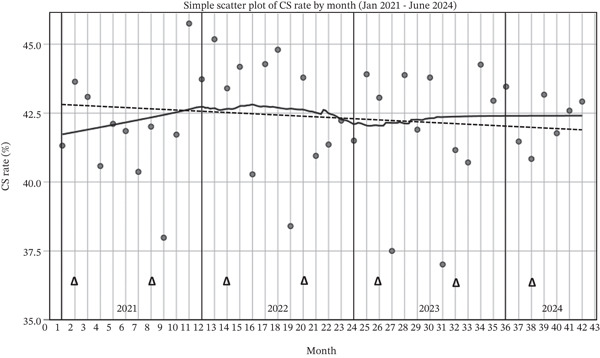
A scatter plot of overall monthly CS rate with linear (dotted line) and LOESS (solid line) regression (*Δ* denotes audit and feedback).

During the study period, the monthly CS rate was assessed to evaluate whether there was a changing trend. The results are demonstrated in a scatter plot with LOESS regression as shown in Figure 1. Overall, the CS rate slightly increased during 2021, slightly decreased during 2022, and was relatively stable during 2023 and 2024 at approximately 43%. A linear regression was also performed, and the results showed only a minimal decrease in CS over the study period from an estimated 35% with the regression coefficient of −0.01% per month.

**Figure 2:**
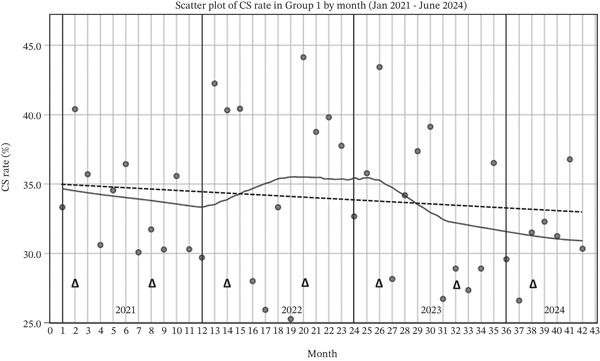
A scatter plot of monthly CS rate in Group 1 with linear (dotted line) and LOESS (solid line) regression (*Δ* denotes audit and feedback).

A trend in CS rate in Group 1 was displayed in Figure 2. The LOESS regression line showed that the CS rate slightly decreased during 2021 and then slightly increased during 2022. However, a more obvious decrease was observed from 2023 to 2024, and the rate was relatively stable at approximately 32%. A linear regression also showed a minimal decrease in CS over the study period from an estimated 35% with a regression coefficient of −0.06% per month.

The percentage of months that the CS rate was achieved at a certain level in each year was evaluated, and the results are displayed in Table 3. For overall CS rate, the rate was < 40% in 7 months (16.7%), 40%–45% in 32 months (76.2%), and > 45% in 3 months (7.1%). For Group 1, the CS rate was < 30% in 13 months (31.0%), 30%–40% in 26 months (61.9%), and > 40% in 3 months (7.1%). It should be noted that in 2023, a CS rate of < 30% was achieved in 6 months (50%), which was double compared with those in 2021 and 2022 (3 months, 25%). In addition, the rate of achievement of the CS rate of 30%–40% consecutively decreased from 2021 to 2023 (75%, 58.3%, and 41.7%, respectively).

**Table 3:** Percentage of month that achieves CS rate at certain level.

CS rate	Year				All
	2021	2022	2023	January–June 2024	
Overall					
< 40%	2 (16.7%)	3 (25%)	2 (16.7%)	0 (0)	7 (16.7%)
40%–45%	9 (75%)	7 (58.3%)	10 (83.3%)	6 (100%)	32 (76.2%)
> 45%	1 (8.3%)	2 (16.7%)	0 (0)	0 (0)	3 (7.1%)
Group 1					
< 30%	3 (25%)	3 (25%)	6 (50%)	1 (16.7%)	13 (31.0%)
30%–40%	9 (75%)	7 (58.3%)	5 (41.7%)	5 (83.3%)	26 (61.9%)
> 40%	0 (0%)	2 (16.7%)	1 (8.3%)	0 (0)	3 (7.1%)

## 4. Discussion

Between 2021 and June 2024, the overall CS rate was 42.9% (95% CI: 42.1%–43.8%). Although the rate was relatively high, it was lower than what was reported from the same institution in 2018 of 48.9%, [18] and lower than reports from other tertiary care hospitals in Thailand of 45%–55%. [20–21] The rate was also higher than the WHO′s previous estimates for Thailand from preceding years of 31%–42%. [2, 3, 5, 6]

Although women in Group 5 (previous CS) contribute the most to overall CS each year, as of current practice in Thailand, women with previous CS will always receive repeat CS, so this group will not be the primary group whose CS rate is supposed to be reduced. The main focus, therefore, was on women in Group 1 who also had a high rate of CS contribution.

The linear regression results showed that the overall CS rate was relatively stable during the study period, with a decrease rate of only 0.01% per month, as shown in Figure 1. The CS rate of Group 1 showed a slightly faster decrease with the rate of 0.06% per month, as shown in Figure 2. The CS rate in Group 1 did not change significantly during the study period at approximately 33.5%. This is, however, lower than a previous report from the same hospital in 2018, which was 37.1%. [18] Nevertheless, a decreasing trend of CS rate in Group 1 was observed during 2023–2024 as shown in Figure 2. The results also revealed that CS rate in Group 1 between 30%–40% was attained in 61.9% of the 42 months. In 2023, the CS rate of < 30% was doubled compared with those in 2021 and 2022. The observed trend was in contrast with a previous report from another university hospital in the southern part of Thailand, which reported that the overall CS rate and CS rate in Group 1 remained high at approximately 55% and 40%, respectively, throughout 2014–2016. [20]

Although there was no strong supporting evidence, the observed declining trend in CS rate, particularly among women in Group 1, could possibly be attributed partly to various interventions that have been implemented on a consistent basis over the past several years. The interventions were developed, modified, and implemented in accordance with the WHO and ACOG recommendations. [10, 11, 13, 14] Routine educational interventions for pregnant women have been modified and updated to incorporate the information on route of delivery into the content. As the majority of CS occurred during labor, modification of the intrapartum care guidelines has been introduced. [11, 13, 14] Compliance with the changes has been regularly assessed through direct observations and audit systems, and these interventions have been regularly reinforced among in‐training residents. Although attending residents initially made decisions on CS, a second opinion from the attending staff was mandatory as a standard practice in our institution, which was in line with the recommendation by the WHO. [10] In addition, an audit and feedback system has also been initiated and is being implemented regularly as recommended by the WHO. [10] The system helps in strengthening other interventions by monitoring the compliance and identifying specific rooms for improvements and solutions. However, further investigations are warranted to evaluate the effects of these combined interventions on the change in CS rate in the future.

Our setting is a tertiary‐care university hospital that some considerable proportion of women with pregnancy complications were included, which may contribute to the high CS rate. A previous study from the same hospital examined the influence of preeclampsia on CS rate and found that preeclampsia contributed to only 10.8% in Group 1, and when preeclampsia was excluded, only a modest change was observed in the overall and group‐specific CS rate [19] However, other contributions of other common pregnancy complications have not yet been evaluated. Being a tertiary‐care hospital could also explain the significant increase in proportions of women in Group 10 as these cases were more likely to have some pregnancy complications [19], whereas many others were referred cases.

Women with private services were excluded from the study to eliminate the confounding effects on CS rate. It has consistently been reported that those receiving private services had an abnormally high CS rate [22–24] due to many reasons including the fact that their caring obstetricians were less likely to comply with the existing guidelines and decisions on CS were usually unfulfilled with obstetric indications, both for prelabor and intrapartum CS. Other related issues might include better time management and unofficial financial incentives. Exclusion of these cases would reflect the actual performance of the institution on CS rate and would also be better in the evaluation of interventions. The issue of private services should be taken into account when evaluating the CS rate, particularly in areas with a high rate of such practices, as is common in Thailand.

Strengths of this study were that a large number of pregnant women were included over a 42‐month period which enabled evaluation of any changing trend. Data on Robson classification were routinely collected prospectively after delivery of each woman, making the information valid and reliable. However, generalization of the results might be limited due to differences in clinical practices and guidelines between hospitals, differences in levels of care provided, and the exclusion of women with private services in this study. In addition, the effectiveness of interventions that were implemented gradually before and continued during the study period on CS rate could not be determined. As recommended by the WHO, the use of Robson classification will generally help health care facilities to evaluate the situation of the use of CS and plan appropriate strategies to optimize CS rate as the objective of the current study. Other determinants and predictive factors for CS, such as maternal demographic characteristics, comorbidities, and so forth, were not evaluated. Future research is needed to determine such predictive factors, both for overall and group‐specific CS, taken into account for other potential confounders, using appropriate statistical models.

Future, long‐term, prospective studies are needed to investigate the issues of changing in CS rate, not only to evaluate quality and performance of care but also to examine the effects and contributions of various interventions to reduce CS. Due to great variations in the contexts of care between hospitals in Thailand, CS rate monitoring should be performed and context‐specific interventions should be developed in each health care facility nationwide.

## 5. Conclusion

During a 42‐month period, a relatively stable high CS rate of 42.9% was observed in a university‐based tertiary care hospital in Thailand, with the highest contribution from women in Groups 1 and 5 of the Robson classification. CS rate in Group 1 showed a notable decrease during 2023–2024 and was relatively stable at approximately 32%. Further monitoring of CS rate and effects of the interventions should be evaluated in the future.

## Funding

No funding was received for this manuscript.

## Disclosure

The author confirms sole responsibility for the study conception and design, data collection, analysis and interpretation of results, and manuscript preparation.

## Conflicts of Interest

The author declares no conflicts of interest.

## Data Availability

The data that support the findings of this study are available from the corresponding author upon reasonable request.
